# Controlling the Secondary Surface Morphology of Electrospun PVDF Nanofibers by Regulating the Solvent and Relative Humidity

**DOI:** 10.1186/s11671-018-2705-0

**Published:** 2018-09-12

**Authors:** Bilal Zaarour, Lei Zhu, Chen Huang, Xiangyu Jin

**Affiliations:** Engineering Research Center of Technical Textiles, Ministry of Education, College of Textiles, Donghua University, No. 2999 North Renmin Road, Songjiang, Shanghai, 201620 China

**Keywords:** Electrospinning, PVDF nanofibers, Secondary surface morphology, Phase separation, Oil clean up

## Abstract

This work presents a simple and reliable method for directly generating polyvinylidene fluoride (PVDF) nanofibers with secondary surface morphology (e.g., porous surfaces, rough surfaces, grooved surfaces, and interior porosity) by using single/binary solvent systems and relative humidity. We clarified the mechanisms responsible for the formation of these morphologies by systematically exploring the molecular interactions among the polymer, solvent(s), and water vapor. Our results proved that the formation of secondary surface morphology needed the presence of water vapor, a non-solvent of the polymer, at an appropriate level of relative humidity. The formation of secondary surface morphology was dependent on the speed of evaporation of the solvent(s) (ACE, DMF, and their mixtures), as well as the inter-diffusion and penetration of the non-solvent (water) and solvent(s). The results of N_2_ physical adsorption-desorption isotherms showed that the macro-porous fibers (> 300 nm) exhibited the highest specific surface area of 23.31 ± 4.30 m^2^/g and pore volume of 0.0695 ± 0.007 cm^3^/g, enabling the high oil absorption capacities of 50.58 ± 5.47 g/g, 37.74 ± 4.33 g/g, and 23.96 ± 2.68 g/g for silicone oil, motor oil, and olive oil, respectively. We believe this work may serve as guidelines for the formation of different structures of macro-porous, rough, and grooved nanofibers with interior porosity through electrospinning.

**Electronic supplementary material:**

The online version of this article (10.1186/s11671-018-2705-0) contains supplementary material, which is available to authorized users.

## Background

Electrospinning is a fiber formation method that involves electrostatic forces for ejecting and stretching polymer jets into fibers. The process currently produces fibers with a diameter ranging from few nanometers to several micrometers [1]. Various morphologies of electrospun fibers including beaded fibers [2], porous fibers [3], grooved fibers [4], multichannel fibers [5], ribbon fibers [6], side-by-side fibers [7], hollow fibers [8], hierarchical fibers [9], rice grain-shaped nanocomposites [10], butterfly wings fibers [11], core-sheath fibers [12], and crimped fibers [13] can be formed by controlling electrospinning parameters [14].

Electrospun nanofibers have shown excellent properties such as high specific surface area, flexibility, ease of functionality, variety of morphology and structure, superior directional strength, and high porosity which makes them a preferred material form for different applications such as energy harvesting [15], sensors [16], filtration [17–19], biomedical applications [20], self-cleaning surfaces [21–23], etc. Studies have demonstrated that by regulating the secondary morphologies (e.g., porous surfaces, grooved surfaces, rough surfaces, and interior porosity) of electrospun fibers, their properties and behavior could be greatly enhanced or changed. For instance, porous fibers have shown extensive use in a wide variety of applications such as catalysis, filtration, and biomedical research due to the increase in their specific surface areas through the introduction of intrafiber pores [24]. Rough fibers have been used to improve the electrical output of the scavenging energy devices due to increasing of the friction areas [25]. Grooved fibers have presented a great potential in the area of tissue engineering and superhydrophobic surfaces [26]. Moreover, increasing the specific surface area and porosity leads to the enhanced performance of absorption [27, 28], catalysis [29, 30], etc.

Previously, we have reported the production of polystyrene fibers with tunable macro-pore structures and distributions by using a microfluidic nozzle containing three channels that allows for liquid mixing from two input channels and synchronized electrospinning of the resulting mixture from the other output channel [3]. Furthermore, we have reported the fabrication of cellulose acetate butyrate and polystyrene fibers with a grooved structure via electrospinning using a mixed solvent system consisting of a high boiling point solvent and a low boiling point solvent [4, 31].

In this study, we demonstrate the fabrication of polyvinylidene fluoride (PVDF) nanofibers with the macro-porous, rough, and grooved surface structures and interior pores using electrospinning without involving any special collection method or post-spinning treatment. Here, PVDF was selected as the model because it can be dissolved in different solvents.

To the best of our knowledge, so far, no studies have been systematically investigated maneuvering the formation of the macro-porous (> 300 nm), rough, and grooved electrospun PVDF nanofibers with internal porosity by controlling the relative humidity. Herein, we reported the electrospinning of PVDF solutions at four levels of relative humidity (5%, 25%, 45%, and 65%) using both single and binary solvent systems. The main objective of this work is to investigate the feasibility of fabricating macro-porous, rough, and grooved fibers with solid and porous interior structures using different levels of relative humidity, and to discover their formation mechanisms. By systematically investigating the effect of the relative humidity on the secondary surface morphology of electrospun PVDF fibers, we concluded that the relative humidity plays an important role in determining the surface and the internal morphology of PVDF fibers. This study can provide useful guidelines for the preparation of secondary surface structure of nanofibers through electrospinning.

## Methods

### Chemicals and Materials

PVDF pellets (Mw = 275,000) were purchased from Sigma-Aldrich, Inc. Acetone (ACE) and N,N-dimethylformamide (DMF) were purchased from Shanghai Chemical Reagents Co., Ltd., Shanghai, China. All materials were used without further purification.

### Electrospinning of PVDF Fibers with Secondary Surface Morphology

In order to obtain macro-porous, rough, and grooved fibers, 18% ACE (*w*/*v*) PVDF solution, 35% DMF (*w*/*v*) PVDF solution, and 25% (ACE/DMF) (*w*/*v*) PVDF solutions at the solvent ratios of (4:1, 2:1, 1:1, 1:2, and 1:4) were prepared, respectively, and each solution was loaded into a plastic syringe. In this work, the solvent ratio was the volume ratio, and the solution concentration was weight/volume (*w*/*v*) (g/ml). A 21 gauge syringe needle was used as the spinneret, which was fixed on a syringe pump (KDS 100, KD Scientific Inc., USA) connected to a high-voltage supplier (Tianjin Dongwen Co., Ltd., China). A grounded drum collector (40 cm in length and 20 cm in diameter) was placed 18 cm away from the spinneret, and the rotating speed was set at 2 rpm to obtain randomly oriented fibers. All the experiments were carried out at 20 °C under different levels of relative humidity (5%, 25%, 45%, and 65%). The temperature was adjusted by the laboratory central air conditioning system and the relative humidity was controlled by the environmental humidity, which could be further set with a narrow window (± 2%) by using a humidifier/dehumidifier. All samples were prepared at feeding rate and applied voltage of 1.5 ml/h and 18 kV, respectively. All previous parameters were adjusted to get fibers with different morphologies and similar diameters.

### Ternary Phase Diagram

The cloud point curves were determined by the titration method at the relative humidity of 65%. PVDF solutions were prepared by dissolving the polymer in the single solvent systems using ACE and DMF, and binary solvent systems using ACE/DMF at the solvent ratio of 1:1. The acquired homogeneous solutions were titrated with deionized water as a non-solvent. At the beginning of permanent turbidity, the solutions composition and the amount of the non-solvent used were noted and plotted in the ternary phase diagram, which was used to represent the binodal curves [32, 33].

### Characterization

The surface morphology and cross-section of the electrospun PVDF nanofibers were checked under field-emission scanning electron microscopy (FE-SEM) (S-4800, Hitachi Ltd., Tokyo, Japan) after gold coating. Cross-sections of the fibers were prepared by placing them in liquid nitrogen and breaking them manually. Fiber diameter was measured using image analysis software (Adobe Acrobat X Pro 10.1.2.45) according to the SEM images. N_2_ physical adsorption-desorption isotherms (JW-BK132F, Beijing Science and Technology Co., China) were measured to determine the specific surface area, pore distribution, and total pore volume.

### Oil Absorption

The capacity of oil absorption was measured at 25 °C using the following method. Then, 15 mL of water–oil mixture with a ratio of 1:1 was prepared and put in a beaker. Further, 0.3 g of the sorbent was added to the beaker to absorb oil for 1 h, and then the wet sorbent was moved to a screen mesh and drained for ~  40 min to ensure that no oil droplets remained on the sorbent. The capacity of oil absorption was calculated according to the following equation:

$$ Q\kern0.5em =\kern0.5em \frac{m_0-{m}_1}{m_1} $$

where *Q* is the capacity of oil absorption (g/g), *m*_0_ is the total mass of the wet sorbent after oil absorption drained for ~  40 min (g), and *m*_1_ is the mass of the sorbent before absorption (g).

## Results and Discussion

To explore the effect of the relative humidity on the secondary morphology of electrospun PVDF fibers, 18% (*w*/*v*) PVDF solution with ACE, 35% (*w*/*v*) PVDF solution with DMF, and 25% (*w*/*v*) PVDF solutions with different ACE/DMF ratios were electrospun.

### Fibers Electrospun from ACE

**Fig. 1:**
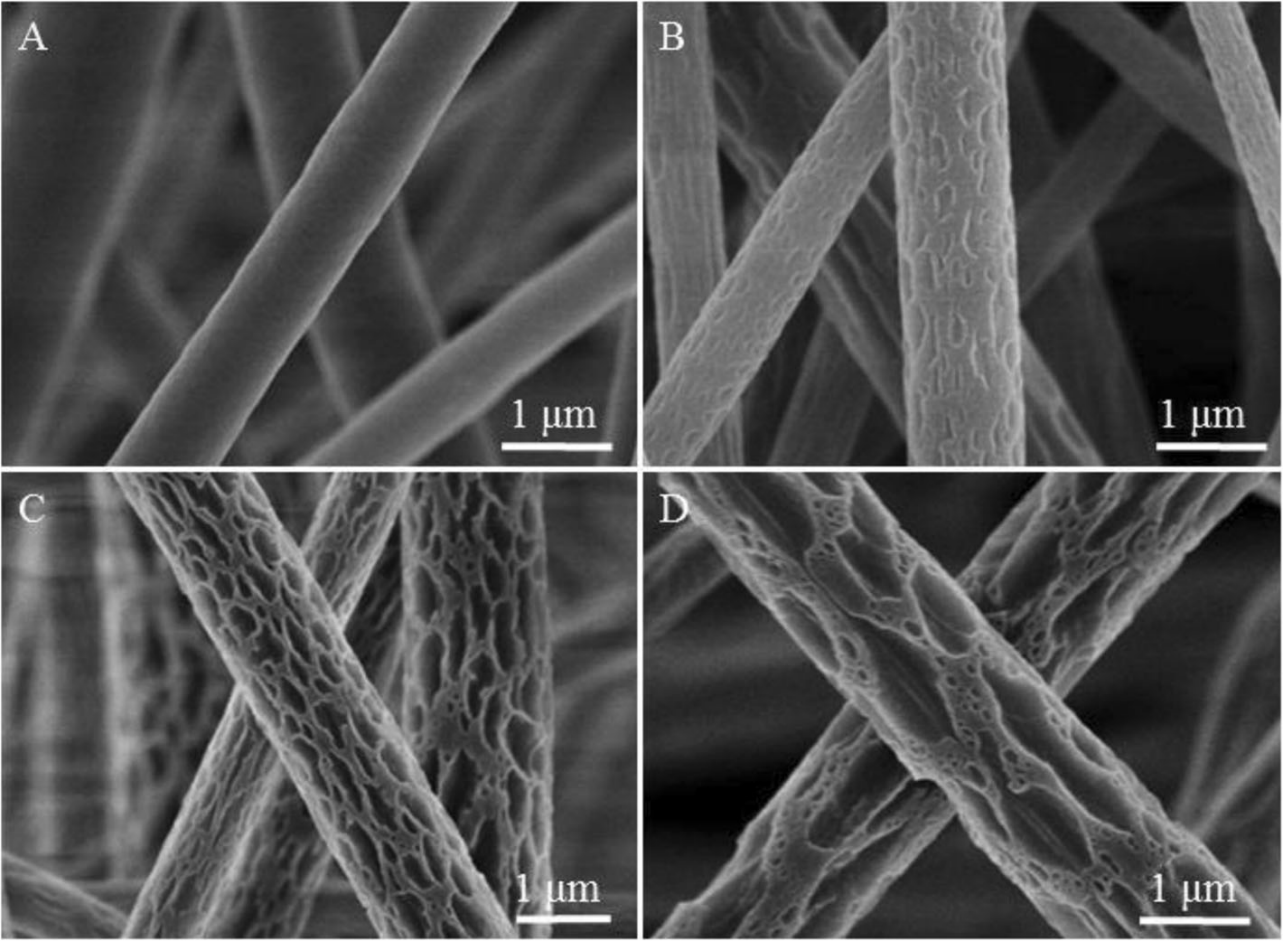
Representative SEM images of samples fabricated by electrospinning 18% (*w*/*v*) PVDF solution from ACE at different levels of relative humidity. **a** 5%, **b** 25%, **c** 45%, and **d** 65%

**Fig. 2:**
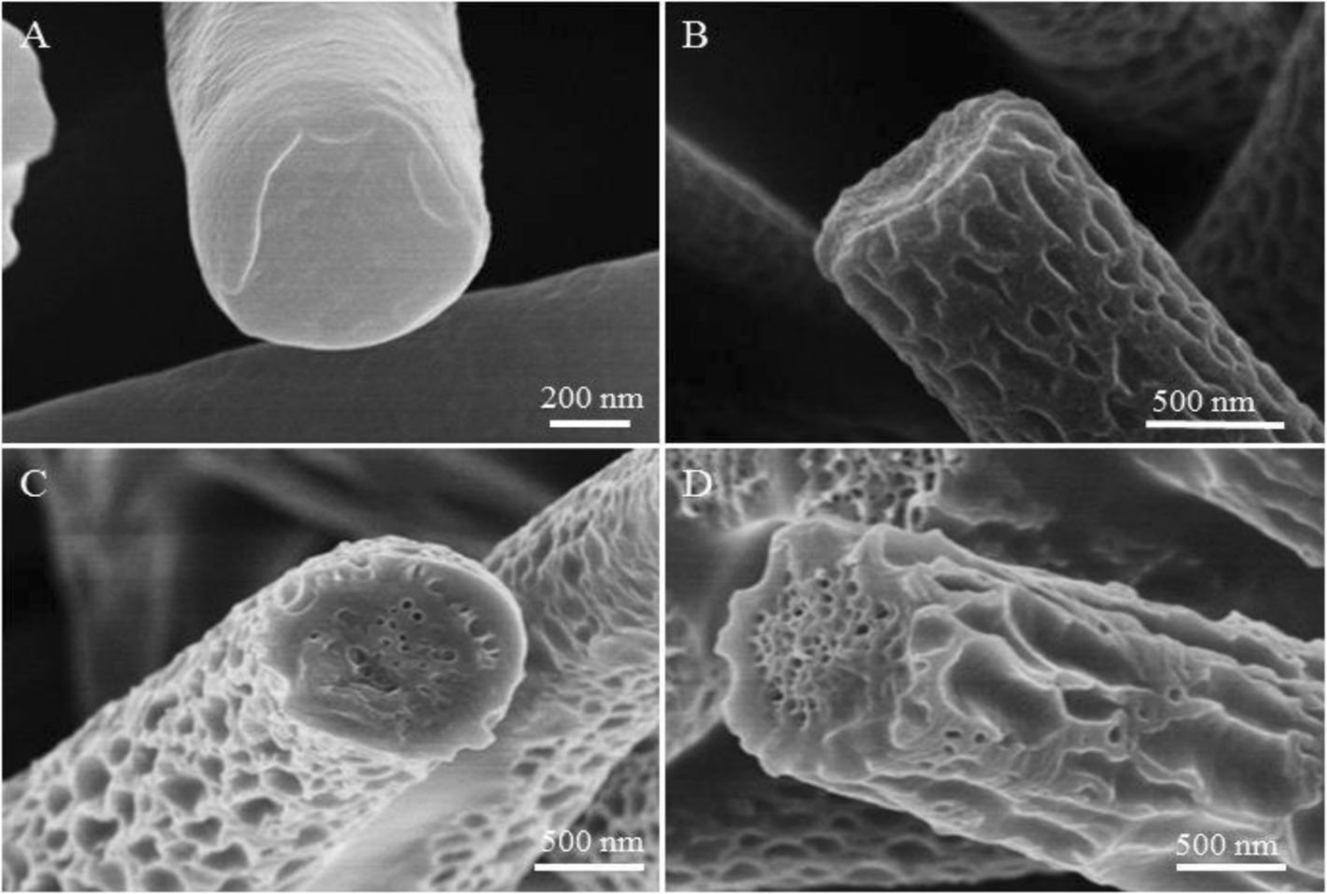
Cross-sectional SEM images of samples fabricated by electrospinning 15% (*w*/*v*) PVDF solution from ACE at different levels of relative humidity. **a** 5%, **b** 25%, **c** 45%, and **d** 65%

Fibers obtained from PVDF/ACE solution at different levels of relative humidity have been exhibited and compared (Figs. 1 and 2). Smooth fibers were formed using PVDF/ACE solution at the relative humidity of 5% (Fig. 1a and Additional file 1: Figure S1A), while macro-porous fibers were produced at the relative humidity of 25%, 45%, and 65% (Fig. 1b–d and Additional file 1: Figure S1B-D). The formation of surface pores should be ascribed to thermal-induced phase separation (TIPS) [24].

**Fig. 3:**
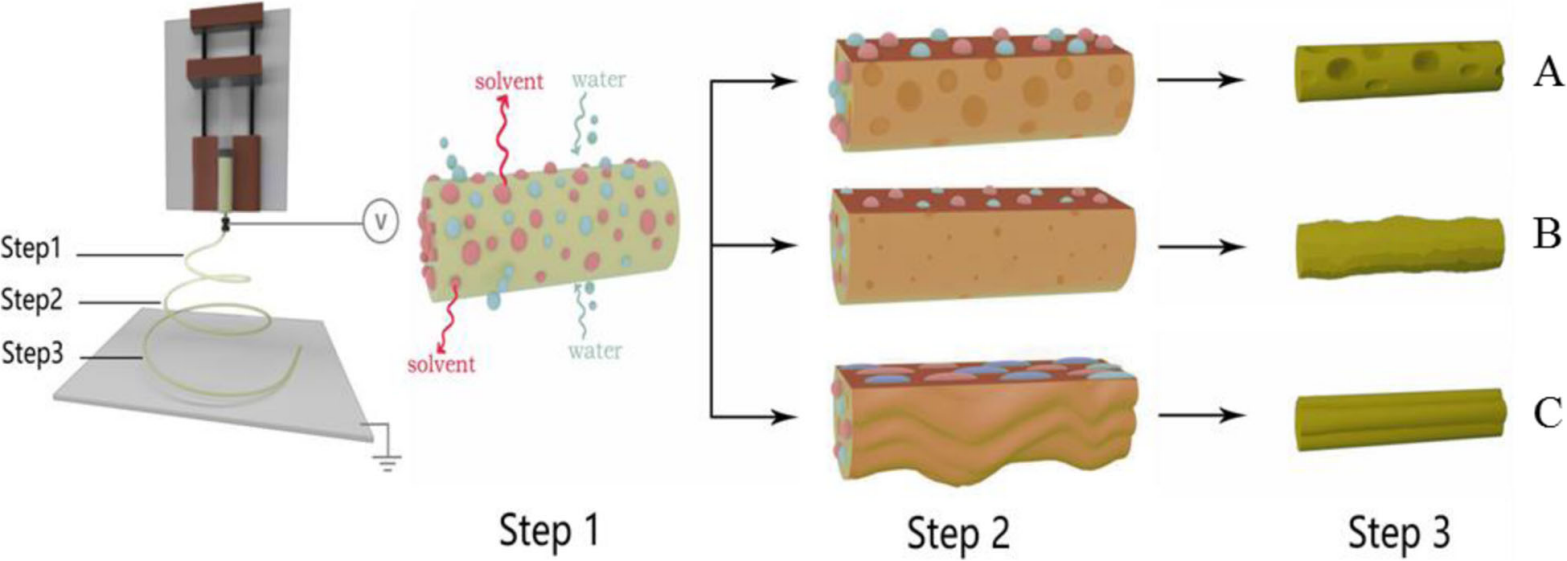
Process diagrams of the solution jet during electrospinning at high relative humidity. Step 1: the evaporation of the solvent and water condensation, step 2: the penetration of water droplets and generation of pores, and step 3: elongating and solidification of fibers. **a** Macro-porous fibers, **b** rough fibers, and **c** grooved fibers

To confirm the formation mechanism of PVDF macro-porous fibers formed from PVDF/ACE solution, we checked the cross-section of fibers formed at different levels of the relative humidity studied. We found that at the relative humidity of 5% and 25%, fibers formed had a solid interior (Fig. 2a, b), while fibers with interior pores were formed at the relative humidity of 45% and 65% (Fig. 2c, d). Thus, we can conclude that fibers formed with a smooth surface and solid interior at the relative humidity of 5% because of no phase separation. While macro-porous fibers with solid interior were formed due to TIPS. Whereas macro-porous fibers with interior pores were formed at the relative humidity of 45% and 65% due to the coexistence of both TIPS and vapor-induced phase separation (VIPS). In other words, when the high volatile solvents evaporated, they absorbed a great amount of heat and thus cooled the surface of the fibers, which led them to condense and attract water droplets on the fibers surface. When the relative humidity increased, the evaporation rate of water droplets decreased, leading to coalescence between the droplets formed macro droplets whose mechanism is known as the nucleation growth (NG) [34]. After the condensed macro water droplets dried, they formed macro-pores on the surface of the fibers. Whereas, the part of water droplets that penetrated the fiber eventually dried to form interior pores. The formation mechanism of the macro-porous fibers at high relative humidity is demonstrated in Fig. 3a. At high relative humidity, the evaporation rate of the water droplets condensed on the fibers surface decreased, giving these droplets more time for merging together due to NG. Therefore, the size of macro-pores on the surface of fibers increased from ~ 50 nm at the relative humidity of 25%, to ~ 100 nm at the relative humidity of 45%, and to ~ 400 nm at the relative humidity of 65%. All surfaces and internal morphologies obtained from PVDF/ACE solution at different levels of relative humidity are summarized in Table 1. Importantly, increasing the relative humidity from 5 to 65% leads to the increase of fibers diameter from ~ 0.77 to ~ 1.81 μm (Additional file 1: Figure S2A).

**Table 1:** Summary of the morphologies of the PVDF fibers and their interiors obtained in this work at different levels of relative humidity and polymer solutions

Relative humidity	Polymer solution	Surface structure	Interior structure
5%	25% (ACE/DMF)(4:1)	Smooth	Solid
	25% (ACE/DMF)(2:1)	Smooth	Solid
	25% (ACE/DMF)(1:1)	Smooth	Solid
	25% (ACE/DMF)(1:2)	Smooth	Solid
	25% (ACE/DMF)(1:4)	Smooth	Solid
	18% ACE	Smooth	Solid
	35% DMF	Smooth	Solid
25%	25% (ACE/DMF)(4:1)	Grooved	Porous
	25% (ACE/DMF)(2:1)	Rough	Porous
	25% (ACE/DMF)(1:1)	Rough	Porous
	25% (ACE/DMF)(1:2)	Smooth	Porous
	25% (ACE/DMF)(1:4)	Smooth	Porous
	18% ACE	Porous	Solid
	35% DMF	Rough	Porous
45%	25% (ACE/DMF)(4:1)	Grooved	Porous
	25% (ACE/DMF)(2:1)	Grooved	Porous
	25% (ACE/DMF)(1:1)	Rough	Porous
	25% (ACE/DMF)(1:2)	Rough	Porous
	25% (ACE/DMF)(1:4)	Rough	Porous
	18% ACE	Porous	Porous
	35% DMF	Rough	Porous
65%	25% (ACE/DMF)(4:1)	Grooved	Porous
	25% (ACE/DMF)(2:1)	Grooved	Porous
	25% (ACE/DMF)(1:1)	Grooved	Porous
	25% (ACE/DMF)(1:2)	Rough	Porous
	25% (ACE/DMF)(1:4)	Rough	Porous
	18% ACE	Porous	Porous
	35% DMF	Rough	Porous

### Fibers Electrospun from DMF

Herein, 35% (*w*/*v*) PVDF solution were electrospun at different levels of relative humidity (5%, 25%, 45%, and 65%).

**Fig. 4:**
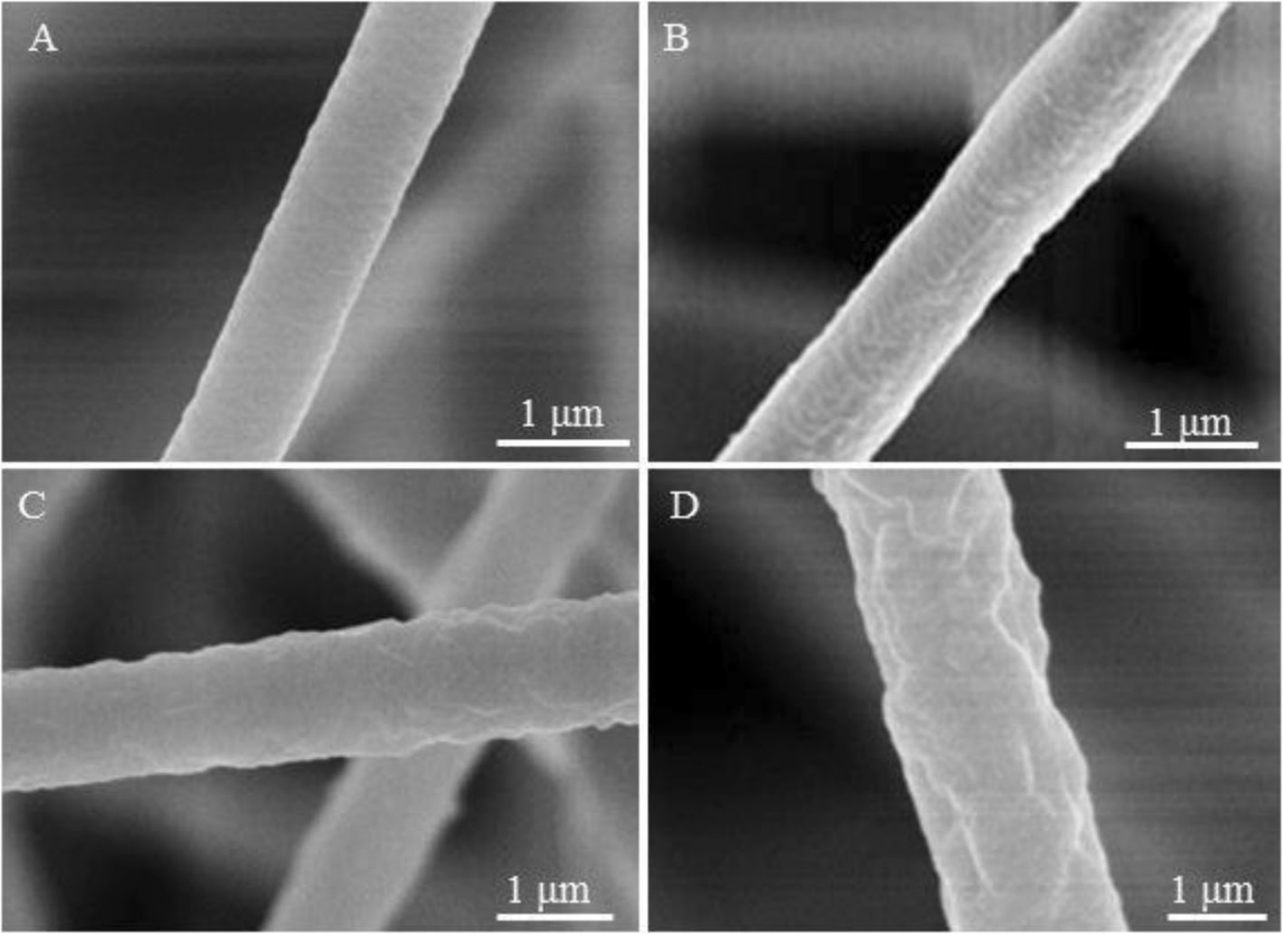
Representative SEM images of samples fabricated by electrospinning 35% (*w*/*v*) PVDF solution from DMF at different levels of relative humidity. **a** 5%, **b** 25%, **c** 45%, and **d** 65%

**Fig. 5:**
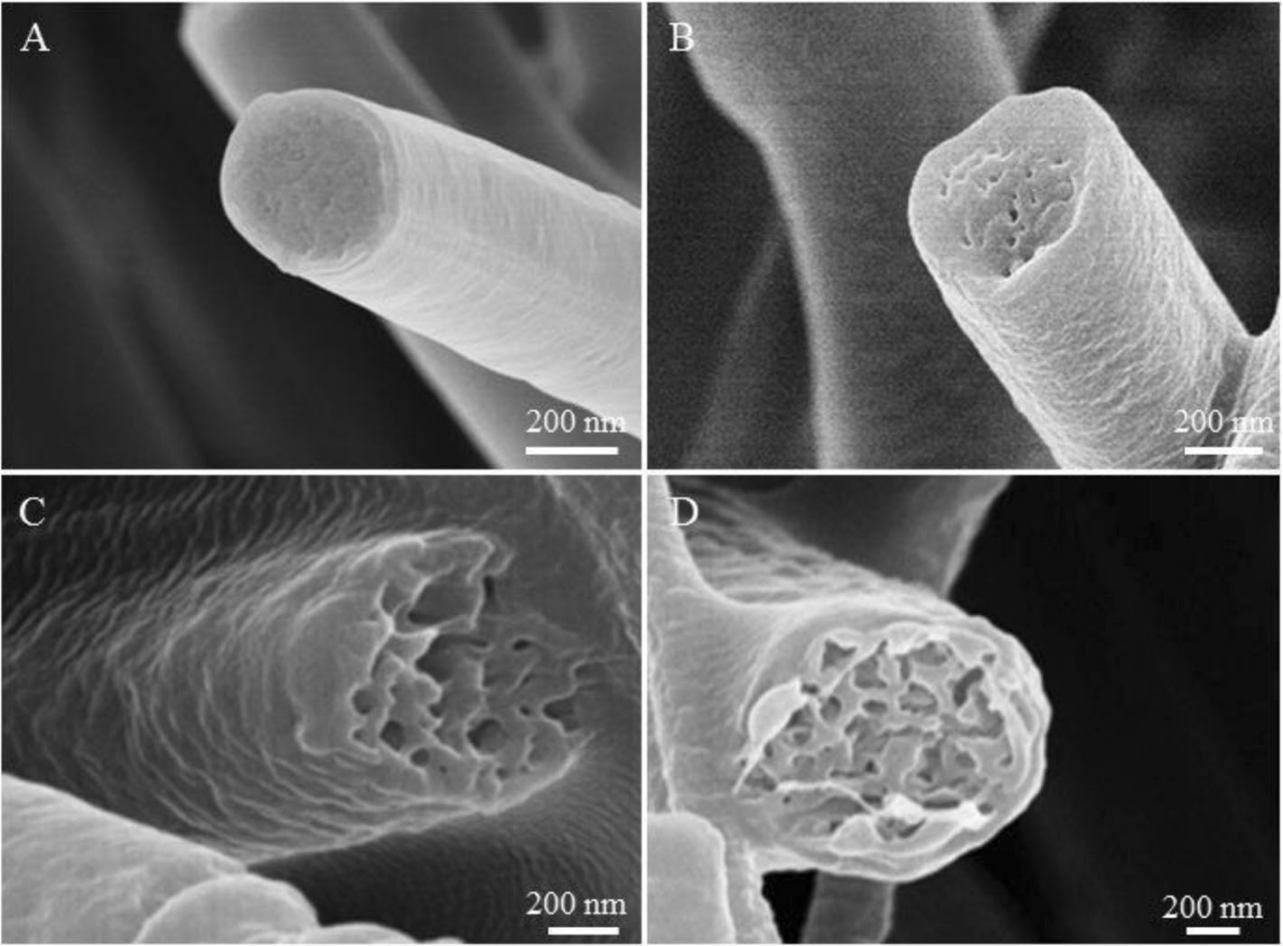
Cross-sectional SEM images of samples fabricated by electrospinning 35% (*w*/*v*) PVDF solution from DMF at different levels of relative humidity. **a** 5%, **b** 25%, **c** 45%, and **d** 65%

Smooth fibers were produced using PVDF/DMF solution at the relative humidity of 5% (Fig. 4a and Additional file 1: Figure S3A), while rough fibers were formed at the relative humidity of 25%, 45%, and 65% (Fig. 4b–d and Additional file 1: Figure S3B-D) due to buckling instability [35] and stretching by electrical force [26]. According to the cross-section of fibers formed at previous relative humidity studied, we found that fibers with solid interior were obtained only at the relative humidity of 5% (Fig. 5a), while fibers with interior pores were formed at the relative humidity of 25%, 45%, and 65% (Fig. 5b–d). In this case, we can say that the fibers formed with a smooth surface and solid interior because of no phase separation, while fibers obtained with a rough surface and interior pores because of VIPS [24]. In other words, the corporate diffusion and penetration of DMF and water vapors played an essential role in forming fibers with interior pores; because of the fact that vapor pressure of water (2.34 kPa) is higher than that of DMF (0.36 kPa) at the temperature of 20 °C, it is reasonable to assume that the water vapor saturated the nearby region of the interface between the air and the jet first, then followed by its action as a non-solvent to precipitate a sheath of PVDF on the surface of the liquid jet. The solidified PVDF layer helped ensnare the DMF inside and slacken its evaporation rate, which possibly banned the water vapor from fast condensing or accumulating on the surface to form big droplets. The water vapor penetrated the sheath and constantly entered the PVDF-DMF phase, resulting in a rapid phase separation. Figure 3d illuminates the formation mechanism of rough fibers at high relative humidity. All surfaces and internal morphologies obtained from PVDF/DMF solution at different levels of relative humidity are summarized in Table 1. Interestingly, the increase of the relative humidity from 5 to 65% leads to the increase of fibers diameter from ~ 0.8 to ~ 1.79 μm (Additional file 1: Figure S2B).

### Fibers Electrospun from ACE/DMF Mixture Solution

**Fig. 6:**
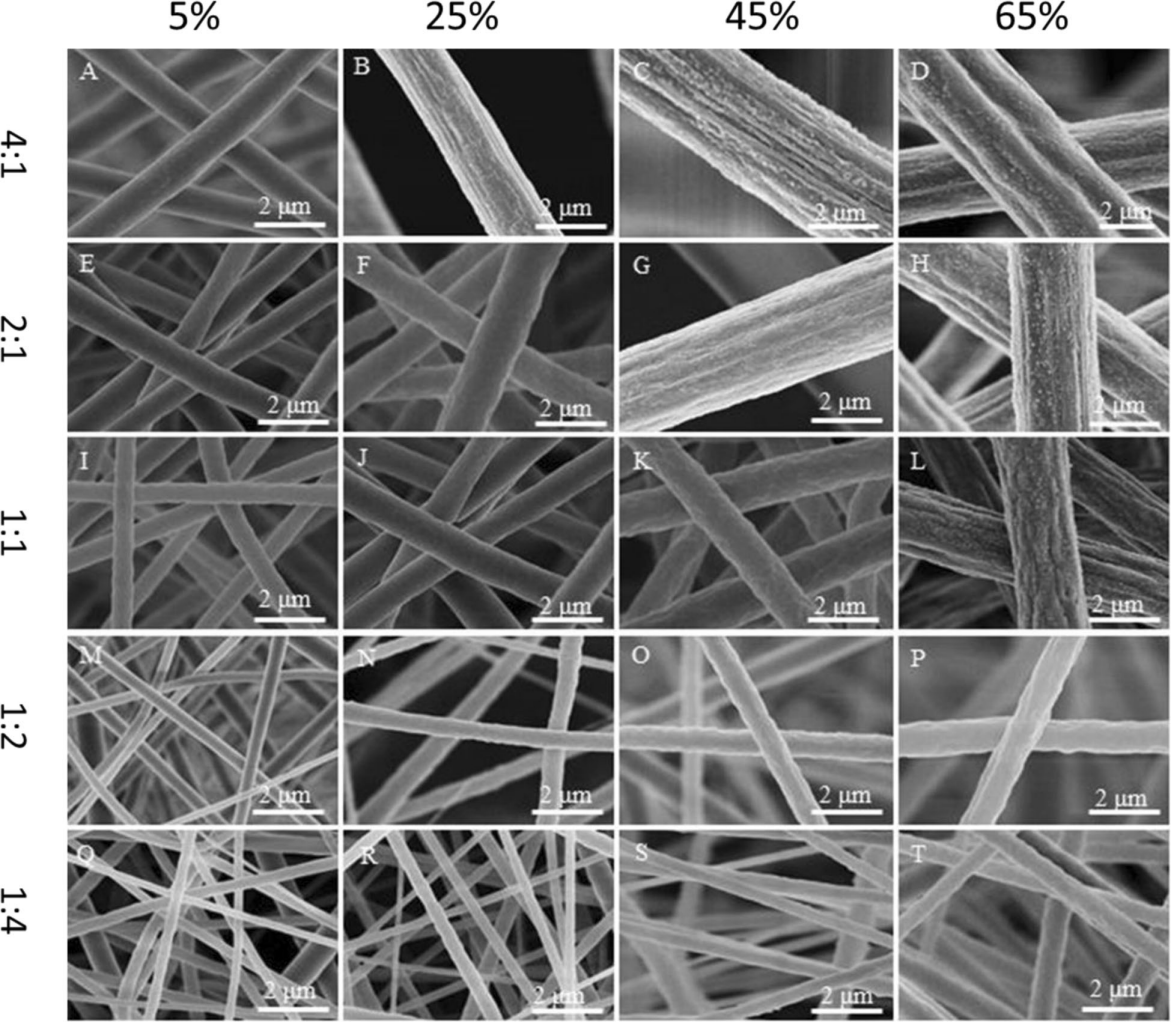
Representative SEM images of samples fabricated by electrospinning 25% (*w*/*v*) PVDF solutions from ACE/DMF at different levels of relative humidity (5%, 25%, 45%, and 65%) and solvent ratios. **a**–**d** 4:1, **e**–**h** 2:1, **i**–**l** 1:1, **m**–**p** 1:2, and **q**–**t** 1:4

In this case, 25% (*w*/*v*) PVDF solutions with different ACE/DMF ratios (4:1, 2:1, 1:1, 1:2, and 1:4) were electrospun at different levels of relative humidity. For 25% (*w*/*v*) (ACE/DMF at the solvent ratio of 4:1), smooth fibers were formed at the relative humidity of 5% (Fig. 6a and Additional file 1: Figure S4A), pillar shallow longitudinal grooved fibers were produced at the relative humidity of 25% (Fig. 6b and Additional file 1: Figure S4B), and pillar longitudinal grooved fibers were produced at the relative humidity of 45% and 65% (Fig. 6c, d and Additional file 1: Figure S4C, D). For 25% (*w*/*v*) (ACE/DMF at the solvent ratio of 2:1), smooth fibers were formed at the relative humidity of 5% (Fig. 6e and Additional file 1: Figure S4E), rough fibers were fabricated at the relative humidity of 25% (Fig. 6f and Additional file 1: Figure S4F), shallow longitudinal pillar grooved fibers were obtained at the relative humidity of 45% (Fig. 6g and Additional file 1: Figure S4G), and pillar longitudinal grooved fibers were produced at the relative humidity of 65% (Fig. 6h and Additional file 1: Figure S4H). For 25% (*w*/*v*) (ACE/DMF at the solvent ratio of 1:1) at the relative humidity of 5%, smooth fibers were observed (Fig. 6i and Additional file 1: Figure S4I), rough fibers were produced at the relative humidity of 25% and 45% (Fig. 6j, k and Additional file 1: Figure S4J, K), and pillar small grooved fibers were produced at the relative humidity of 65% (Fig. 6l and Additional file 1: Figure S4L). For 25% (*w*/*v*) (ACE/DMF at the solvent ratios of 1:2 and 1:4), when the relative humidity ≤ 25%, smooth fibers were seen (Fig. 6m, n, q, r and Additional file 1: Figure S4M, N, Q, R), whereas rough fibers were produced at the relative humidity of 45% and 65% (Fig. 6o, p, s, t and Additional file 1: Figure S4O, P, S, T).

**Fig. 7:**
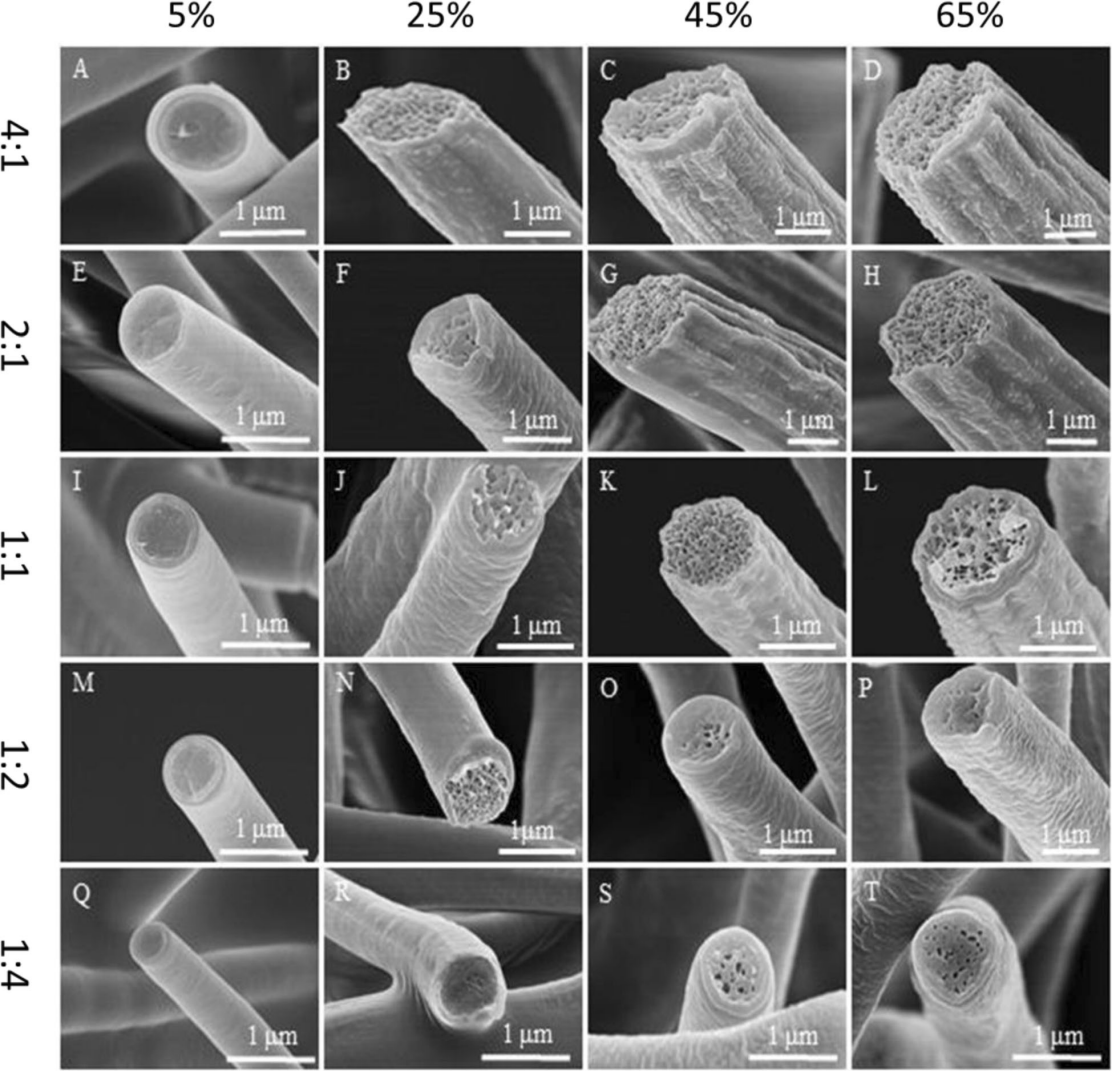
Cross-sectional SEM images of samples fabricated by electrospinning 25% (*w*/*v*) PVDF solutions from ACE/DMF at different levels of relative humidity (5%, 25%, 45%, and 65%) and solvent ratios. **a**–**d** 4:1, **e**–**h** 2:1, **i**–**l** 1:1, **m**–**p** 1:2, and **q**–**t** 1:4

To be more accurate about the formation mechanism of the PVDF grooved fibers formed from ACE/DMF, we checked the cross-section of fibers formed at all the solvent ratios and different levels of the relative humidity studied. We noticed that at the relative humidity of 5%, all fibers formed had solid interior. Herein, we conclude that no phase separation happened at the formation of fibers with smooth surface and solid interior (Fig. 7a, e, i, m, q). At the relative humidity of 25%, 45%, and 65%, all fibers produced had interior pores. Grooved fibers with interior pores were fabricated by wrinkle-based elongation mechanism [36]. In this case, due to the fast evaporation of the highly volatile ACE (vapor pressure, 24 kPa) and phase separation, a glassy skin was formed at the early stage of electrospinning, subsequently the wrinkled surface of the jet was formed because of the formation of the interior pores, and elongated into grooved fibers afterwards (Fig. 7b–d, g, h, l). Figure 3c explains the formation mechanism of the grooved fibers at high relative humidity. The formation of nanopillars on the surface of the grooved fibers might be due to the fact that the ACE trapped in the fibers by the glassy skin, which faced a fast evaporation of ACE, but some weak points might still exist, resulting in the formation of nanopillars.

Fibers with rough surfaces and interior pores were formed due to VIPS (Fig. 7f, j, k, o, p, s, t). Fibers with smooth surfaces and interior pores were also formed due to VIPS (Fig. 7n, r) [24, 37]. It is worth mentioning that the width and depth of the grooves increased by increasing the relative humidity. All surfaces and internal morphologies obtained from ACE/DMF mixture solution at different levels of relative humidity are concluded in Table 1. We noticed that increasing the relative humidity from 5 to 65% leads to the increase of fibers diameter from ~ 1 to ~ 3.75 μm, ~ 0.85 to ~ 2.9 μm, ~ 0.6 to ~ 2 μm, ~ 0.35 to ~ 1 μm, and ~ 0.26 to ~ 0.7 μm for the following solvent ratios of 4:1, 2:1, 1:1, 1:2, and 1:4, respectively (Additional file 1: Figure S2C-G).

**Fig. 8:**
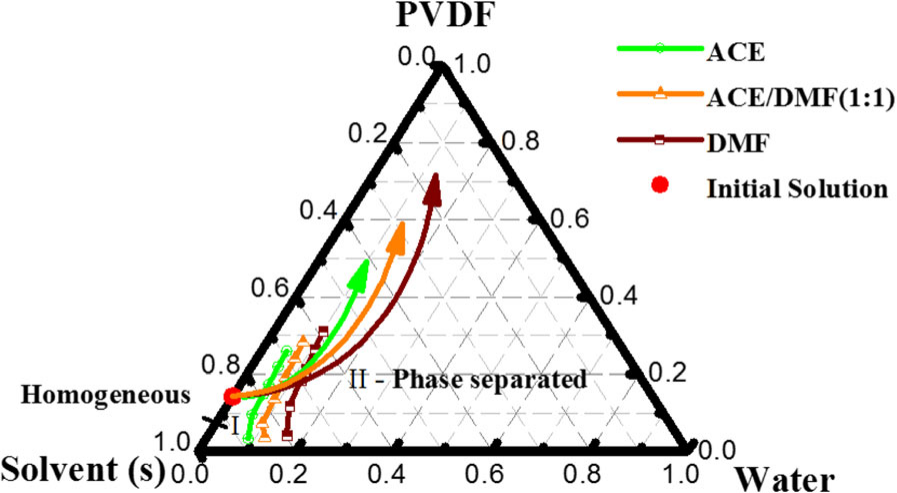
Phase diagram of PVDF, solvent (s), and water at the relative humidity of 65%. The red point refers to the initial solution

Due to the importance of the high relative humidity in forming secondary surface structures of the PVDF fibers, we illustrated the phase behavior of electrospinning solutions by creating a phase diagram at the relative humidity of 65% (Fig. 8). The diagram is divided into two zones by a binodal curve. The solution jet is cloudless and homogeneous upon being extruded from the spinneret (zone I). With the high volatilization rate of ACE, low volatilization rate of DMF, and the subsequent permeation of water into solution jet, the proportion of the components (PVDF, solvent (s), and water) in the jet is dynamically altered to follow the path shown by the arrows. The solution jet starts to enter zone II, after crossing the bimodal curve, where it turns into cloudy and separates into multiphase due to the thermodynamic instability [37, 38]. A higher volatile solvent (ACE) is represented by a steeper arrow, which corresponds to a faster happening of phase separation.

**Fig. 9:**
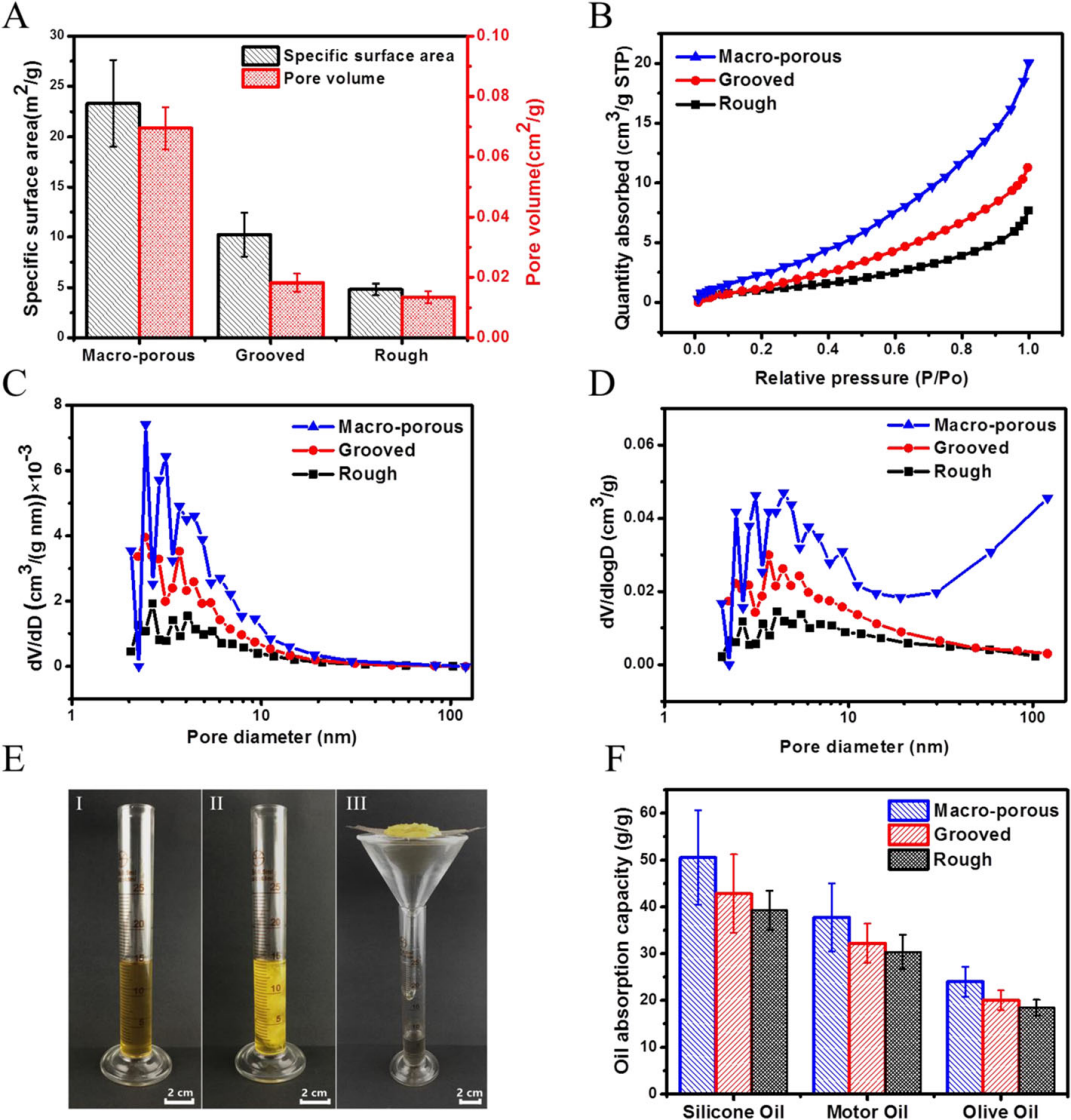
Characterizations of the macro-porous, grooved, and rough fibers. **a** Specific surface area and pore volume. **b** The nitrogen adsorption isotherms. **c** dV/dD—pore diameter curve. **d** dV/dlogD—pore diameter curve. **e** Pictures of oil absorption. (I) 15 mL of water-oil mixture (1:1) without sorbent, (II) during absorption, (III) during draining. **f** Oil absorption capacities

In order to quantify the surface area and pore structure of the fibers, the nitrogen adsorption isotherms of the macro-porous fibers (Fig. 1d), rough fibers (Fig. 4d), and grooved fibers (Fig. 6l) having similar diameters were chosen for comparison. The specific surface areas of the macro-porous, grooved, and rough fibers were 23.31 ± 4.30 m^2^/g, 10.26 ± 2.19 m^2^/g, and 4.81 ± 0.58 m^2^/g, and the pore volumes were 0.0695 ± 0.007 cm^3^/g, 0.0182 ± 0.003 cm^3^/g, and 0.0135 ± 0.002 cm^3^/g, respectively (Fig. 9a). These results coordinated with the maximal nitrogen adsorption of the macro-porous, grooved, and rough fibers which were 20.06 cm^3^/g, 12.29 cm^3^/g, and 7.49 cm^3^/g, respectively (Fig. 9b). We further confirmed that mesopores (2–50 nm) existed in the macro-porous, grooved, and rough fibers (Fig. 9c), whereas macro-pores (> 100 nm) only appeared in the macro-porous fibers, resulting in their high specific surface area and pore volume (Fig. 9d).

Since PVDF is a hydrophobic but not an oleophobic material, the PVDF sorbents can absorb oil while repelling water. We next demonstrated the application of the macro-porous, grooved, and rough fibers for oil absorption (Fig. 9e). Three typical oils (silicon oil, motor oil, and olive oil) were selected to check the different samples.

The typical properties of these oils are listed in Table 2. As expected, among the three kinds of oil absorption materials, the macro-porous fibers showed the highest oil absorption capacity of 50.58 ± 5.47 g/g, 37.74 ± 4.33 g/g, and 23.96 ± 2.68 g/g for silicon oil, motor oil, and olive oil, respectively (Fig. 9f). Particularly, the macro-porous fibers exhibited 1.18, 1.17, and 1.19 times oil absorption capacities of the grooved fibers for silicon oil, motor oil, and olive oil, respectively. Moreover, the macro-porous fibers exhibited 1.29, 1.24, and 1.26 times oil absorption capacities of the rough fibers for silicon oil, motor oil, and olive oil, respectively. These results should be attributed to the fact that the macro-porous fibers have the highest surface area, while the rough fibers have the lowest surface area among all the samples. Among the three types of oil studied, all tested samples exhibited the best absorption capacity for silicon oil, possibly because of the higher viscosity of the silicon oil.

**Table 2:** The basic properties of the oils used in this work

Oil types	Density (g/cm^3^)	viscosity (mPa s)	Surface tension (mN/m)
Olive oil	0.9112	81.5	33.075 ± 0.030
Motor oil	0.8581	135.4	30.246 ± 0.030
Silicon oil	0.9549	468.3	19.979 ± 0.030

## Conclusions

We have demonstrated an appropriate and reliable method for the formation of macro-porous, rough, and grooved PVDF nanofibers with internal pores. In order to understand the mechanism responsible for the formation of PVDF fibers, we tested three solvent systems (i.e., ACE, DMF, and ACE-DMF mixture) under different levels of relative humidity (5%, 25%, 45%, and 65%). We discovered that no phase separation occurred at the relative humidity of 5% by using the previous solvents, resulting in the formation of smooth fibers with a solid interior. We found that the formation of macro-pores on the surface of the fibers with solid interior at the relative humidity of 25% should be attributed to TIPS due to the high vapor pressure of ACE and nucleation mechanism, while the formation of macro-pores on the fibers surface with interior pores at the relative humidity of 45% and 65% should be ascribed to the coexistence of both TIPS and VIPS mechanisms. Additionally, we found that the low vapor pressure of DMF played a core role in the production of the rough fibers with pores in the interiors by VIPS. While wrinkle-based elongation mechanism played a key role in fabricating the grooved fibers with a porous interior structure. The macro-porous fibers (> 300 nm) exhibited the highest performance of oil absorption of 50.58 ± 5.47 g/g, 37.74 ± 4.33 g/g, and 23.96 ± 2.68 g/g for silicon oil, motor oil, and olive oil, respectively. Importantly, our understanding of the mechanisms responsible for the formation of the macro-porous, rough, and grooved PVDF fibers with interior porosity can serve as an important reference for the fabrication of electrospun fibers by regulating the solvent and relative humidity.

## Additional File

Additional file 1: **Figure S1.** SEM pictures (lower magnification) of the porous electrospun PVDF fibers. Figure S2 The diameter of fiber. Figure S3 and S4 SEM pictures (lower magnification) of the rough and grooved electrospun PVDF fibers. (DOCX 2656 kb)

## Abbreviations

ACE: Acetone
DMF: N,N dimethylformamide
PVDF: Polyvinylidene fluoride
TIPS: Thermal induced phase separation
VIPS: Vapor-induced phase separation
