# Supplementary material for: Controlling the Secondary Surface Morphology of Electrospun PVDF Nanofibers by Regulating the Solvent and Relative Humidity
Source: Nanoscale Res Lett. 2018 Sep 12;13:285. doi: 10.1186/s11671-018-2705-0 (PMC6135735; doi:10.1186/s11671-018-2705-0)
Supplement: Supplementary file 1 — Figure S1. SEM pictures (lower magnification) of the porous electrospun PVDF fibers. Figure S2 The diameter of fiber. Figure S3 and S4 SEM pictures (lower magnification) of the rough and grooved electrospun PVDF fibers. (DOCX 2656 kb) [file 11671_2018_2705_MOESM1_ESM.docx]

**[Supporting Information]**

**Controlling the Secondary Surface Morphology of Electrospun PVDF Nanofibers by Regulating the Solvent and Relative Humidity**

**Bilal Zaarour ^a^**^†^**, Lei Zhu ^a^**^†^**, Chen Huang ^a^, Xiangyu Jin ^a^***

^a^ Engineering Research Center of Technical Textiles, Ministry of Education, College of Textiles, Donghua University, No. 2999 North Renmin Road, Songjiang, Shanghai 201620, China.

^†^ These authors contributed equally to this work.

^*^ Correspondence: [jinxy@dhu.edu.cn](mailto:jinxy@dhu.edu.cn)

**Author Contributions**

BZ ^†^ and LZ^†^ contributed equally to this work. BZ and XJ conceived the original concept. BZ and LZ designed, conducted the experiments, and analyzed the data. BZ wrote the manuscript. BZ, LZ, CH, and XJ revised the manuscript. All authors have read and approved the final manuscript.


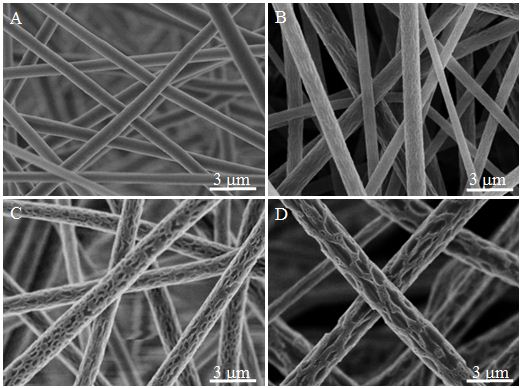


**Fig. S1** Representative SEM images of samples fabricated by electrospinning 18% (w/v) PVDF solution from ACE at different levels of relative humidity. A) 5%, B) 25%, C) 45%, and D) 65%.


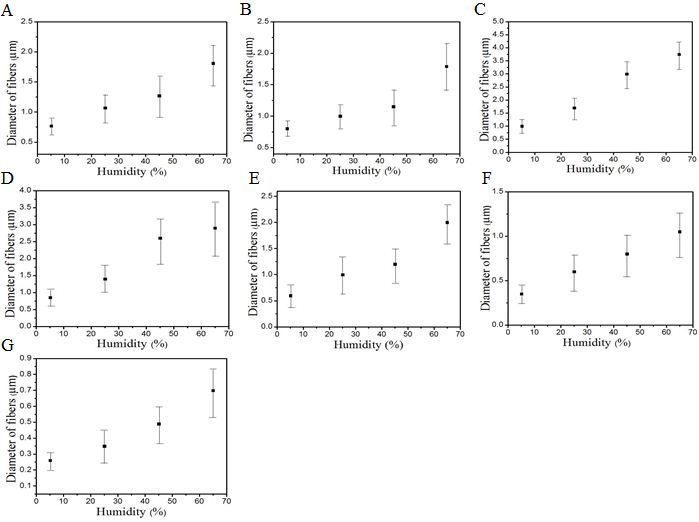


**Fig. S2** The influence of the relative humidity on the diameters of the fibers formed at different solutions. A) 18% (w/v) ACE. B) 35% (w/v) DMF. C-G) 25%(w/v) ACE/DMF at the solvent ratios : C) 4:1, D) 2:1, E) 1:1, F) 1:2, G) 1:4.


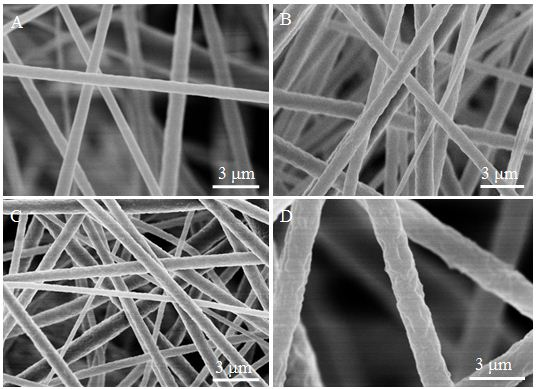


**Fig. S3** Representative SEM images of samples fabricated by electrospinning 35% (w/v) PVDF solution from DMF at different levels of relative humidity. A) 5%, B) 25%, C) 45%, and D) 65%.


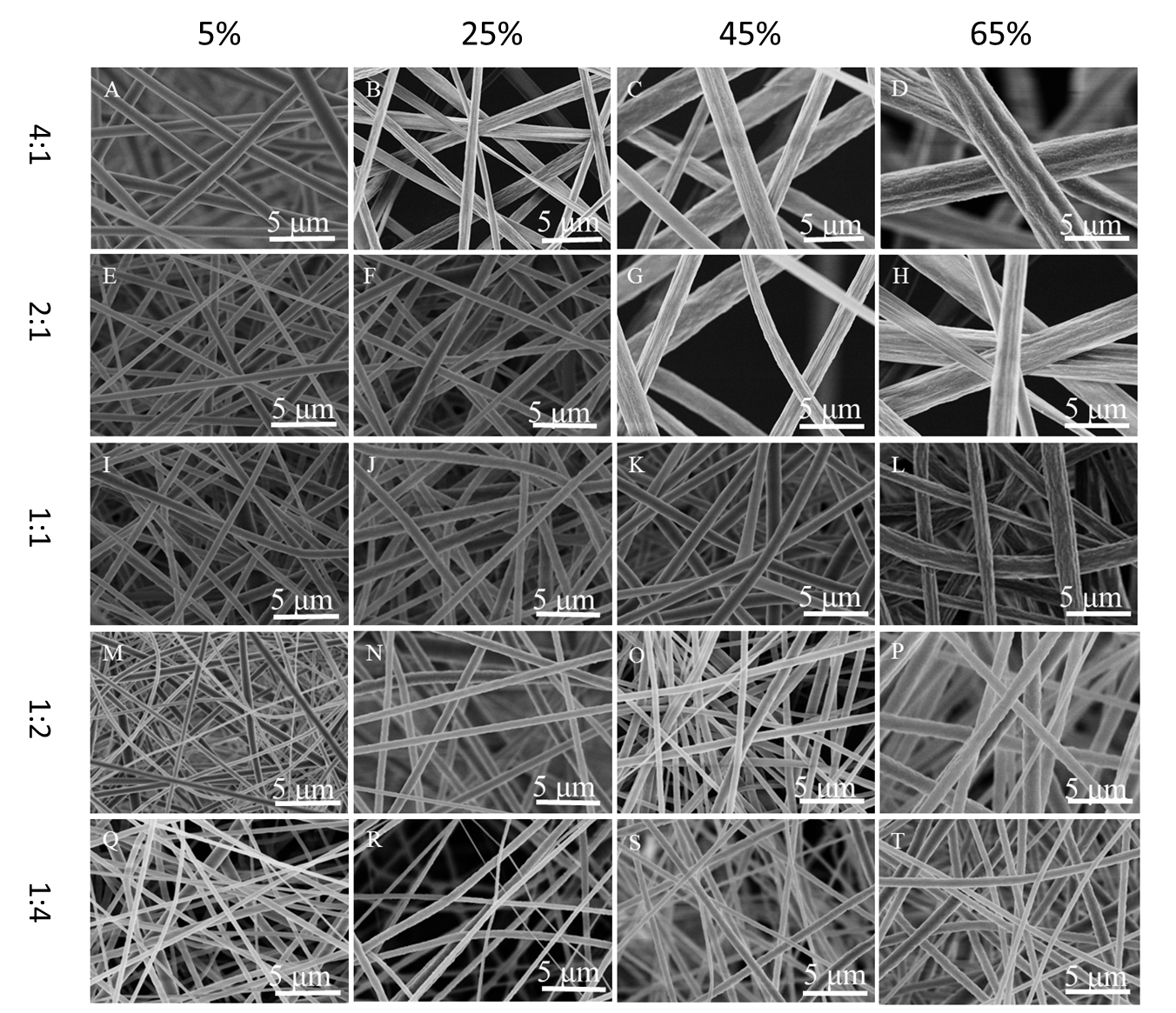


**Fig. S4** Representative SEM images of samples fabricated by electrospinning 25% (w/v) PVDF solutions from ACE/DMF at different levels of relative humidity (5%, 25%, 45%, and 65%) and solvent ratios. A-D) 4:1, E-H) 2:1, I-L) 1:1, M-P) 1:2, Q-T) 1:4.

20 mm

20 mm
